# The Board of Medical Examiners of the State of New York

**Published:** 1890-07

**Authors:** 


### THE BOARD OF MEDICAL EXAMINERS OF THE STATE OF NEW YORK.

On another page of this number of the JOURNAL our readers will find the full text of the law, recently passed, which provides for a state examination of candidates for the practice of medicine in this state, and which directs the manner in which such examination shall be conducted.

We bespeak for the act a careful study, as in our judgment it is easily the most important medical law of this century.

In its enactment the State of New York enters upon a new policy towards the medical profession, for after its day of taking effect, September 1, 1891, the diploma of a medical college, no matter where located, and no matter how attained, ceases to be a license to practice.

By this law the people give notice that they resume the right to go behind the diploma in order to ascertain how much if any of professional ability the said diploma represents. That in taking this important step the state has acted wisely we firmly believe—that the provocation has been serious and of long standing, the literature of medical education in this country for the last quarter of a century abundantly attests that the result will demonstrate the superior efficiency of the new method of licensing physicians we confidently expect.

In the meantime we are glad to congratulate our readers upon the fact that this law was drawn by the committee on legislation of the Erie County Society—that after receiving the unanimous endorsement of that Society at a special meeting held September 8, 1883, it was given to the profession at large through the columns of this journal.

We spoke at that time under the title of Medical Education in the State of New York, as follows: "Ever since the JOURNAL has been under its present editorial management it has been outspoken in exposing the iniquities of our present system of medical education, and we have urged, with all boldness, the application of the only effective remedy, namely: "The complete separation of the unfortunate, unwise, "and debasing co-partnership of the educating and licensing powers. "As a brave step in the right direction, therefore, we rejoice to be able "to call the attention of our readers to the proceedings of the Erie "County Medical Society as published in this issue. The committee "on legislation have well done the duty imposed upon them and "desire the thanks and hearty support of every member of the profes- "sion in the State of New York."

We also will remember our warning to the advocates of this reform to the effect that they need not look for a walk-over, an easy victory, but that they might prepare to meet an astute, powerful and well-organized opposition. The history of the repeated failures of the past seven years in getting the bill passed indicates that we did not over-estimate the power of the enemy.

Perhaps some of our readers will say that this law is very unlike the bill we started out with in 1883. This calls for three boards, that asked for one. To which we would remark that the years of waiting, the failures and the study of this question, has given us a better plan than that of 1883.

And although our sectarian friends are to have an examination board in homeopathy and another in eclectic medicine, yet there is only one Board of Medical Examiners of the State of New York, and that will be composed of seven physicians to be nominated by the Medical Society of the State of New York.

